# Endothelial dysfunction in middle-aged and older men with low testosterone is associated with elevated circulating endothelin-1

**DOI:** 10.1152/ajpregu.00218.2024

**Published:** 2025-01-31

**Authors:** Matthew C. Babcock, Lyndsey E. DuBose, Kerry L. Hildreth, Brian L. Stauffer, Wendy M. Kohrt, Megan M. Wenner, Kerrie L. Moreau

**Affiliations:** 1 Division of Geriatric Medicine, School of Medicine, University of Colorado Anschutz Medical Campus, Aurora, Colorado, United States; 2 Geriatric Research, Educational and Clinical Center, Veterans Affairs Eastern Colorado, Denver, Colorado, United States; 3 Division of Cardiology, School of Medicine, University of Colorado Anschutz Medical Campus, Aurora, Colorado, United States; 4 Division of Cardiology, Denver Health Medical Center, Denver, Colorado, United States; 5 Department of Kinesiology and Applied Physiology, University of Delaware, Newark, Delaware, United States

**Keywords:** Aging, endothelin-1, flow-mediated dilation, testosterone

## Abstract

Low testosterone in middle-aged/older men contributes to accelerated vascular aging, including endothelial dysfunction. However, the mechanisms by which low testosterone affects endothelial dysfunction are not well understood. We sought to determine whether higher endothelin-1 (ET-1) levels are associated with reduced brachial artery flow-mediated dilation (FMD) in middle-aged/older men with low testosterone. Plasma ET-1 was quantified in 60 men categorized as young (*n* = 20, age = 30 ± 4 yr, testosterone = 510 ± 63 ng/dL), middle-aged/older with normal testosterone (*n* = 20, age = 59 ± 6 yr, testosterone = 512 ± 115 ng/dL), or middle-aged/older with low testosterone (*n* = 20, age = 60 ± 8 yr, testosterone = 265 ± 47 ng/dL). Endothelial function was determined via brachial artery FMD. Venous and arterial endothelial cells were harvested via endovascular biopsy in a subset of participants and stained for ET-1 expression. Middle-aged/older men with normal testosterone exhibited lower brachial artery FMD (5.7 ± 2.2%) compared with young men (7.3 ± 1.3%, *P* = 0.020), which was exaggerated in middle-aged/older men with low testosterone (4.0 ± 1.8%, *P* = 0.010 vs. middle-aged/older men with normal testosterone). Plasma ET-1 was not different between young (5.6 ± 0.9 ng/dL) and middle-aged/older men with normal testosterone (6.0 ± 1.4 ng/dL, *P* = 0.681) but was higher in middle-aged/older men with low testosterone (7.7 ± 2.8 ng/dL) compared with both groups (*P* < 0.001 vs. young men; *P* = 0.013 vs. middle-aged/older men with normal testosterone). There was no difference in venous (*P* = 0.616) or arterial (*P* = 0.222) endothelial cell ET-1 expression between groups. There was a significant inverse association between plasma ET-1 and FMD (*r* =−0.371, *P* = 0.004). These data suggest that the accelerated age-associated reduction in endothelial dysfunction in middle-aged/older men with low testosterone is related to higher circulating ET-1.

## NEW & NOTEWORTHY

Middle-aged/older men with low testosterone have reduced vascular endothelial function compared with young and age-matched men with normal testosterone. In this manuscript, we demonstrate that men with low testosterone have higher plasma endothelin-1, which is associated with worse brachial artery flow-mediated dilation. The source of higher plasma endothelin-1 remains unknown; however, higher circulating endothelin-1 appears to be a mechanism contributing to reduced vascular endothelial function in men with low testosterone.

## INTRODUCTION

Advancing age is the primary risk factor for the development of cardiovascular diseases (CVD) and more than 90% of CVDs occur in middle-aged/older adults (1). In men, observational studies consistently report an association between low endogenous serum testosterone and increased CVD risk (2-6), suggesting that low endogenous sex hormone concentrations contribute to elevated CVD risk in middle-aged/older men. Although the link between low testosterone and increased CVD risk has been well established, the mechanisms remain poorly understood. We recently demonstrated that middle-aged/older men with low testosterone have accelerated age-related endothelial dysfunction (i.e., reduced brachial artery flow-mediated dilation, FMD; 7), a key antecedent in the development of CVD, related in part to greater oxidative stress and inflammation. However, these factors only explained a small amount of the variance between low testosterone concentrations and lower FMD in middle-aged/older men, leaving questions about additional factors that may be contributing to worse endothelial function in men with low testosterone.

An additional factor that could contribute to endothelial dysfunction in middle-aged/older men with low testosterone that is also associated with oxidative stress and inflammation is endothelin-1 (ET-1). The ET system is an important regulator of age-associated vascular dysfunction (8, 9) and prior studies have demonstrated a clear influence of sex hormones on the endothelin system (8, 10, 11). In older men, ET-1 concentrations are higher (compared with younger men) and act via endothelin subtype A (ETA) receptors on vascular smooth muscle cells (VSMCs) to increase vasoconstriction and oppose the action of vasodilators such as nitric oxide, subsequently reducing FMD (9, 12, 13). Furthermore, ET-1 can negatively affect nitric oxide bioavailability (14), further reducing endothelial-mediated vasodilation. However, previous reports have not considered the impact of androgen deficiency on plasma or cellular ET-1 concentrations, meaning that the effects of declining testosterone cannot be distinguished from a general effect of aging, per se.

Therefore, we sought to determine whether higher circulating and local ET-1 contributes to accelerated age-associated reductions in endothelial function in middle-aged/older men with low testosterone. We hypothesized that middle-aged/older men with low testosterone would have higher ET-1 concentrations in plasma and vascular endothelial cells compared with younger and age-matched men with normal testosterone and that ET-1 would be associated with worse endothelial function assessed using brachial artery FMD.

## METHODS

### Study Design

These data are from a recently completed clinical trial (ClinicalTrials.gov Identifier NCT02758431) designed to examine the effects of low testosterone on cardiovascular aging in men and, therefore, no women were included. The Colorado Multiple Institutional Review Board approved all study protocols and procedures, which conform to the provisions of the Declaration of Helsinki. All participants provided written and verbal consent before participating. We have previously reported the effects of low testosterone on age-associated reductions in brachial artery FMD (7) in a subset of the participants presented here, however, the influence of low testosterone on ET-1 and associations with brachial artery FMD have not been previously reported.

### Study Participants

Men of all races/ethnic backgrounds aged 18–40 yr (young, *n* = 20) or 50–75 yr (middle-aged/older, *n* = 40) were eligible to participate in this study. Middle-aged/older men were categorized as having either normal (400–1,000 ng/dL, *n* = 20) or low [<300 ng/dL (15), *n* = 20] total testosterone from a fasted venous blood sample collected at screening and confirmed with a second morning fasted blood sample. Participants with total testosterone between 300 and 399 ng/dL were excluded. Men were included in the study only if they met the following criteria: *1*) no use of exogenous sex hormones or testosterone boosters for at least 1 year; *2*) body mass index (BMI) <40 kg/m^2^; *3*) nonsmokers; *4*) resting blood pressure (BP) <160/90 mmHg; *5*) non-diabetic and fasted plasma glucose <126 mg/dL; *6*) free from CVD, cancer, renal, liver, or respiratory disease and healthy as assessed by medical history, physical exam, standard blood chemistries, and electrocardiogram at rest and during a graded exercise treadmill test to fatigue; *7*) sedentary-to-recreationally active (<3 days/wk of vigorous aerobic exercise); *8*) no use of medications that might influence cardiovascular function including antihypertensive and lipid-lowering medications; and *9*) no use of vitamin supplements or anti-inflammatory medications or willing to stop 1 mo before and throughout the study.

### Participant Characteristics

Screening BP was measured in the morning under fasted conditions (water only) and abstinence of caffeine (overnight) and exercise (at least 20 h). BP was measured in both arms via oscillometric assessment (Carescape V100, GE Medical Systems) following ≥10 min of seated rest. BP was measured in triplicate and the average of the higher arm is reported here. Body fat was determined using dual X-ray absorptiometry (Hologic Horizon). Peak oxygen consumption was determined using an incremental treadmill exercise protocol, as reported previously (7).

### Measurements

Participants were required to fast and abstain from caffeine and alcohol for at least 10 h and exercise for ≥20 h before the start of the experimental study visit, which was conducted in the morning between 0700 and 1200 h. Participants rested supine in a dimly lit, temperature-controlled room for ≥15 min before any blood or cell sample collection or data recording.

#### Endothelial cell protein expression.

A subset of participants underwent venous (young, *n* = 15; middle-aged/older with low testosterone, *n* = 17; middle-aged/older with normal testosterone, *n* = 17) and/or arterial (young, *n* = 12; middle-aged/older with low testosterone, *n* = 12; middle-aged/older with normal testosterone, *n* = 14) endothelial cell collections. The procedures used for the collection and measurements of venous and arterial endothelial cells have been described in detail previously (16-21). Briefly, endothelial cells were collected from an antecubital vein or the brachial artery using sterile J-wires advanced (~4 cm beyond the tip of the catheter) and retracted through an 18-gauge (for venous cell collections) or 3.0 French (for arterial cell collections) catheter. The wires were transferred to a dissociation buffer solution, and cells were recovered by washing and centrifugation. Cells were fixed with 3.7% formaldehyde, plated on poly-l-lysine coated slides (Sigma Chemical, St. Louis, MO), and stored at −80°C for future staining.

For immunofluorescence staining, cells were rehydrated with PBS and rendered permeable using 0.1% Triton X-100 (Alfa Aesar, Ward Hill, MA). After blocking nonspecific binding with 5% donkey serum (Jackson ImmunoResearch, West Grove, PA), cells were incubated with monoclonal antibodies for ET-1 (1:200 dilution, Invitrogen No. MA3-005) and von Willebrand factor (vWF) (1:200 dilution, Dako No. A008202-5). Cells were incubated with donkey anti-mouse Alexa Fluor 555 (1:500 dilution, Invitrogen No. A32773) and donkey anti-rabbit Alexa Fluor 488 (1:200 dilution, Invitrogen No. A32790).

Slides were systematically scanned using a fluorescence microscope (Eclipse 80i, Nikon, Melville, NY) to identify endothelial cells [positive staining of von Willebrand factor (vWF)], and nuclear integrity was confirmed using DAPI (4′,6′-diamidino-2-phenylindole hydrochloride) staining. Endothelial cell images were digitally captured using a Photometrics CoolSNAPfx digital camera (Roper Scientific, Inc., Tucson AZ) and analyzed using NIS Elements BR 4.20.02 to quantify the intensity of ET-1 staining (i.e., average pixel intensity). vWF intensity was also quantified as a marker of vascular inflammation (22). Values are reported as ratios of endothelial cell protein expression/human umbilical vein endothelial cell (HUVEC; control cells) protein expression. Reporting ratios minimized the potential confounding factor of differences in intensity of staining among different staining sessions. All staining, imaging, and analysis were performed by the same investigator (MCB).

#### Blood sampling.

ET-1 blood samples were collected in a vacutainer containing ethylenediaminetetraacetic acid (EDTA), and aprotinin and plasma ET-1 concentrations were determined using radioimmunoassay (Peninsula Laboratories, Inc.). As reported previously (7), fasted plasma concentrations of glucose, insulin, total cholesterol (Roche Diagnostic systems, Indianapolis, IN), and high-density lipoprotein (HDL) cholesterol (Diagnostic Chemical Ltd, Oxford CT) were determined using enzymatic/colorimetric methods. Homeostatic model assessment of insulin resistance (HOMA-IR) was calculated as (glucose·insulin)/405 (23). Low-density lipoprotein (LDL) cholesterol was calculated using the Friedewald equation (24). Oxidized LDL was measured using an enzyme-linked immunosorbent plate assay (Alpco Diagnostics, Windham, NH). Serum total antioxidant status (TAS), a measure of overall antioxidant capacity, was measured using an enzymatic kit (Randox Laboratories, Oceanside, CA). Interleukin-6 (IL-6) was measured using an enzyme-linked immunoassay, and high-sensitivity C-reactive protein (CRP) was measured using an immunoturbidimetric method. Total serum testosterone, estradiol, and sex-hormone binding globulin (SHBG) were measured via chemiluminescence using a Beckman Coulter Access II analyzer. Free testosterone was calculated for each participant from concentrations of serum testosterone, SHBG, and albumin using an online algorithm (www.issam.ch) using the Vermeulen equation (25). Testosterone categorizations for middle-aged/older men (i.e., low or normal testosterone) were confirmed by measuring testosterone on at least two occasions. All assays were performed at the University of Colorado Clinical Translational Sciences Institute Clinical Translational Research Center Core laboratory.

#### Brachial artery flow-mediated dilation.

Brachial artery diameter and blood flow velocity measurements were acquired using Doppler ultrasound (Vivid I, GE) with a multifrequency linear-array transducer, as previously described (7, 26-28). Briefly, a cuff was placed around the forearm, and images of the brachial artery and blood flow velocity were acquired. The ultrasound probe was held in place using a stereotactic clamp. Five minutes of forearm occlusion was achieved by inflating the cuff to 250 mmHg and, following cuff release, images of the brachial artery were recorded for 2 min. Brachial artery diameters were coded by number and analyzed by an investigator blinded to group status (KLM) using commercially available software (Vascular Research Tools 6, Medical Imaging Applications, LLC). These procedures conform with published guidelines for assessing vascular endothelial function using brachial artery FMD (29).

### Data and Statistical Analysis

#### Statistical analysis.

The statistical approaches were informed by recent guidelines for statistical reporting of cardiovascular research (30). Normality of distribution for continuous variables was determined qualitatively using histograms and quantitatively using the Shapiro–Wilk test. Normally distributed participant characteristics, sex hormones, FMD, and circulating factors were examined using Student’s *t* test or one-way ANOVAs. In the case of a significant effect, Tukey’s post hoc analysis was performed to examine differences between groups. In the case of non-normally distributed variables (HOMA-IR, Insulin, follicle-stimulating hormone, ET-1, IL-6, white blood cell count, neutrophils, and monocytes), data were natural log transformed to improve distributions. In these cases, data are presented in their original units to improve interpretability. Relations between continuous variables were examined using Pearson’s product-moment correlation coefficient. To gain additional insight into the potential confounding effects of age on these associations, we performed partial correlation analyses between ET-1 and FMD controlling for age and testosterone. Statistical analyses were completed using GraphPad Prism 10.4.0 and IBM SPSS 28.

## RESULTS

### Participant Characteristics

Participant characteristics are reported in Table 1. Middle-aged/older men were older (*P* < 0.001), had higher body mass (*P* < 0.001), BMI (*P* < 0.001), total body fat (*P* < 0.001), blood pressure (*P* < 0.001 for systolic and diastolic BP), total cholesterol (*P* = 0.002) and LDL (*P* = 0.003) cholesterol compared with young men, regardless of gonadal status. Middle-aged/older men with low and normal testosterone were similar in age (*P* = 0.929), body mass (*P* = 0.085), total body fat (*P* = 0.662), systolic (*P* = 1.000) and diastolic (*P* = 0.945) blood pressure, aerobic capacity (*P* = 0.362), total (*P* = 1.000) and LDL (1.000) cholesterol, estradiol (*P* = 0.077), follicle-stimulating hormone (*P* = 0.728), and luteinizing hormone (*P* = 1.000). Middle-aged/older men with low testosterone had higher BMI (*P* = 0.020), triglycerides (*P* = 0.004), blood glucose (*P* = 0.039), insulin (*P* = 0.015), and HOMA-IR (*P* = 0.009) compared with middle-aged/older men with normal testosterone.

### Endothelin-1, Circulating Factors, and Flow-Mediated Dilation

**Figure 1.**
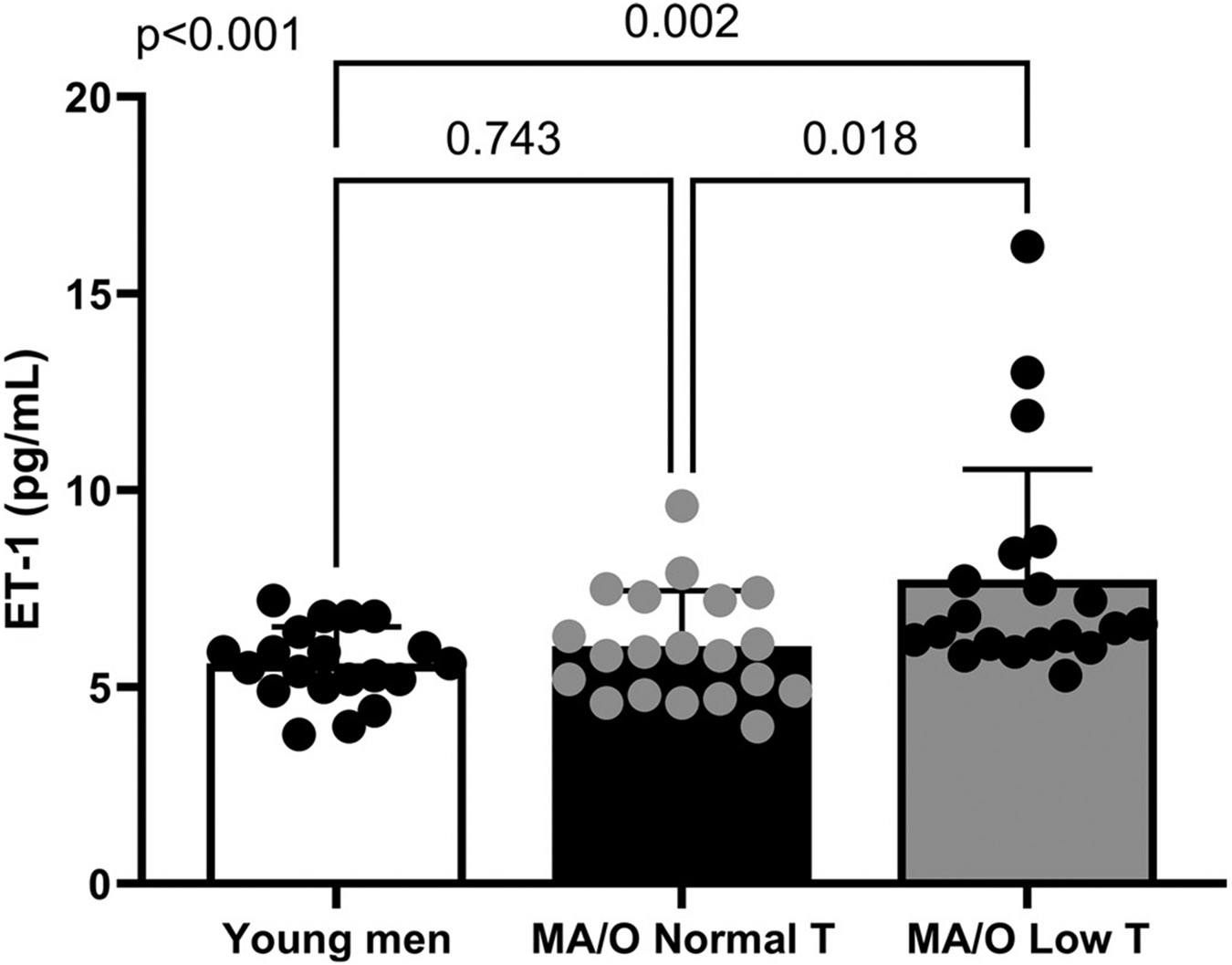
Plasma ET-1 concentrations are significantly higher in middle-aged/older men with low testosterone (*n* = 20) compared with young (*n* = 20) and middle aged/older men with normal testosterone (*n* = 20). Data were not normally distributed and therefore natural log transformed before statistical examination but are presented in their original units to improve interpretability. *P* values reflect the main effect of a one-way ANOVA and post hoc testing. Data are represented as individual data points with means ± SD. ET-1, ET-1, endothelin-1.

There were significant differences in ET-1 concentrations between groups (*P* < 0.001, η^2^ = 0.199). Young and middle-aged/older men with normal testosterone had similar ET-1 concentrations (*P* = 0.743), whereas middle-aged/older men with low testosterone had higher ET-1 compared with both young (*P* = 0.002) and middle-aged/older men with normal testosterone (*P* = 0.018) (Fig. 1). Arterial and venous endothelial cell ET-1 expressions are presented in Table 2 and were not different between groups (arterial endothelial cell ET-1, *P* = 0.222; venous endothelial cell ET-1, *P* = 0.616). In addition, arterial and venous endothelial cell vWF expressions were not different between groups (arterial endothelial cell vWF, *P* = 0.677; venous endothelial cell vWF, *P* = 0.744; Table 2). Other circulating factors can also be found in Table 2. As reported previously (7), markers of oxidative stress (oxidized LDL, TAS) were not different between groups. Middle-age/older men had higher circulating levels of IL-6 (*P* < 0.001) and CRP (*P* = 0.004) compared with young men, regardless of gonadal status. Middle-aged/older men with low testosterone had higher IL-6 and CRP compared with age-matched men with normal testosterone (IL-6, *P* = 0.041; CRP, *P* = 0.004). Similarly, WBCs were increased in middle-aged/older men with low testosterone compared with middle-aged/older men with normal testosterone (*P* = 0.015); however, there were no differences in neutrophils or monocytes between groups.

**Figure 2.**
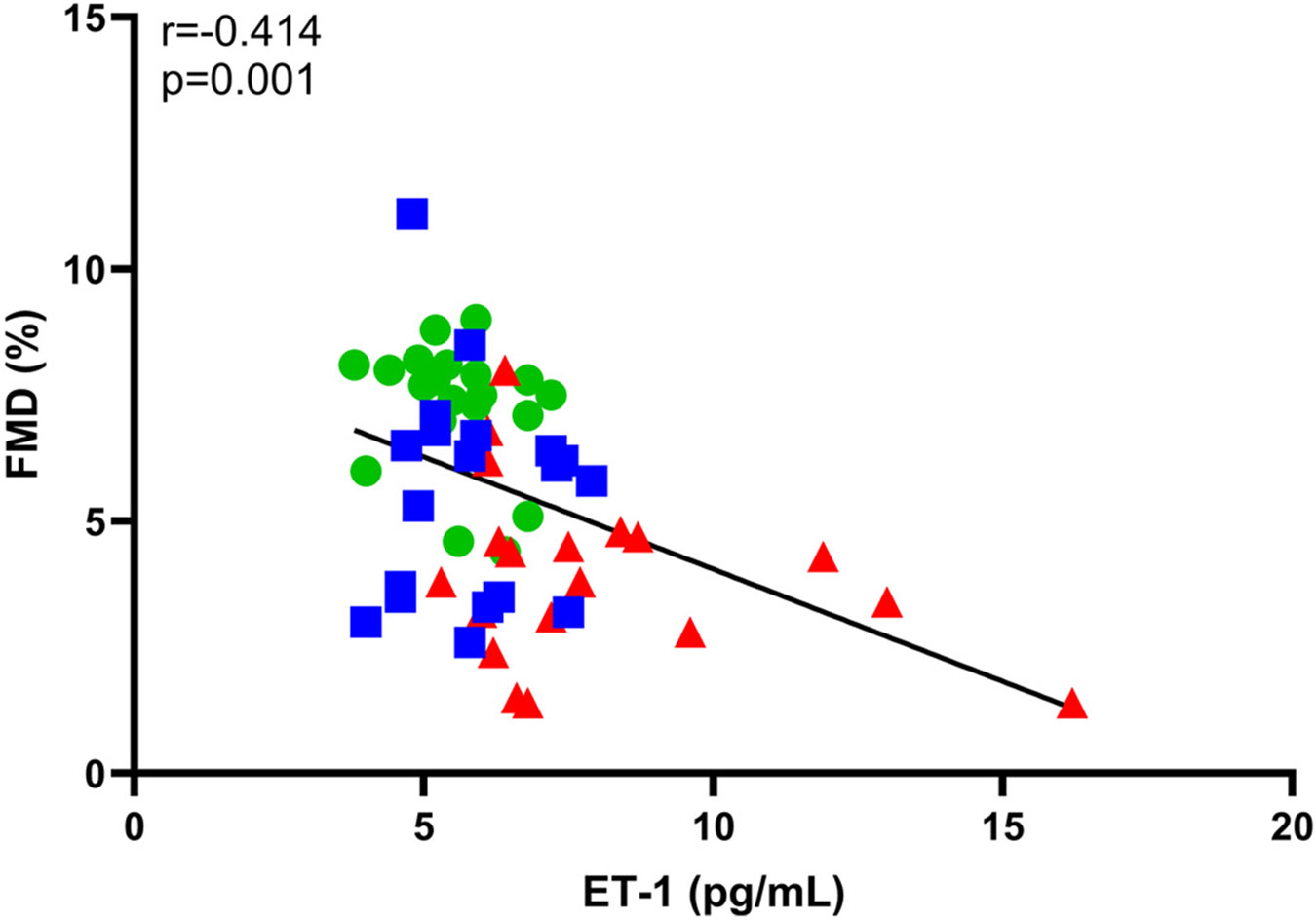
A significant inverse association exists between plasma ET-1 concentrations and brachial artery FMD. Plasma ET-1 concentrations were not normally distributed and therefore natural log transformed before statistical examination but are presented in their original units to improve interpretability. The association between plasma ET-1 and brachial artery FMD was examined using Pearson’s product-moment correlation coefficient. Young men (*n* = 20) are indicated using green circles, middle-aged/older men with normal testosterone (*n* = 20) are indicated using blue squares, and middle-aged/older men with low testosterone (*n* = 20) are indicated using red triangles. ET-1, ET-1, endothelin-1; FMD, flow-mediated dilation.

**Figure 3.**
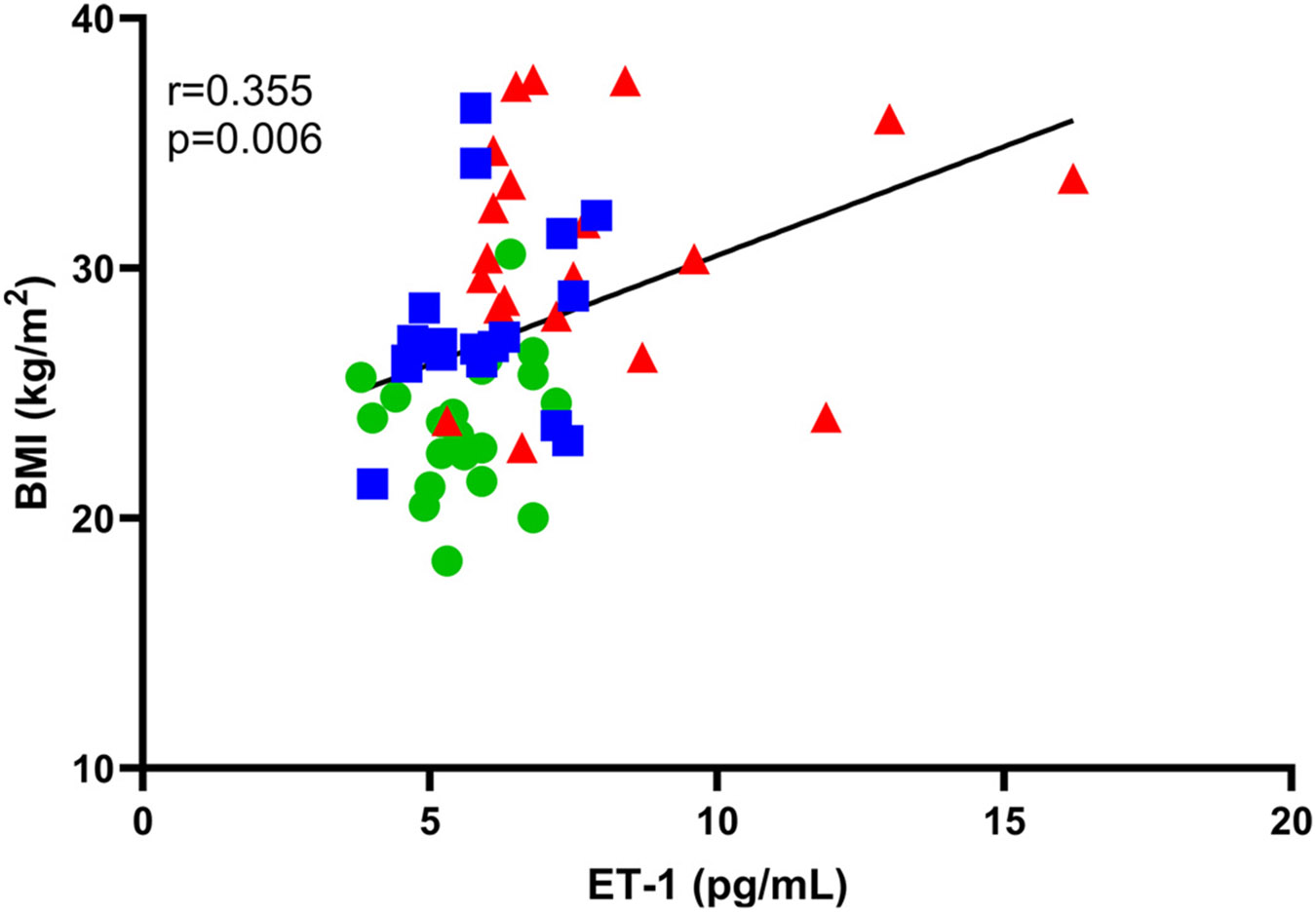
A significant association exists between plasma ET-1 concentrations and BMI. Plasma ET-1 concentrations were not normally distributed and therefore natural log-transformed before statistical examination but are presented in their original units to improve interpretability. The association between plasma ET-1 and BMI was examined using Pearson’s product-moment correlation coefficient. Young men (*n* = 20) are indicated using green circles, middle-aged/older men with normal testosterone (*n* = 20) are indicated using blue squares, and middle-aged/older men with low testosterone (*n* = 20) are indicated using red triangles. BMI, body mass index; ET-1, endothelin-1.

As previously reported, middle-aged/older men with normal testosterone had reduced brachial artery FMD compared with young men; an effect that was exaggerated in middle-aged/older men with low testosterone (Table 3) (7). There was a significant inverse association between natural log-transformed ET-1 concentrations ([lnET-1]) and FMD (Fig. 2). This association was weakened but remained significant when controlling for the effects of age via partial correlation analysis (*r* = −0.272, *P* = 0.039), whereas the association was no longer significant when controlling for testosterone (*r* = −0.198, *P* = 0.136). There was also a significant association between [lnET-1] and BMI (Fig. 3). Partial correlation analyses controlling for age (*r* = 0.275, *P* = 0.035) weakened this association, and controlling for testosterone resulted in an association that was no longer significant (*r* = 0.192, *P* = 0.144). Other correlations are presented in Table 4. There were significant inverse associations between [lnET-1], testosterone, and free testosterone, but no association with other sex hormones or gonadotropins. There were no associations with markers of oxidative stress; however, ET-1 was significantly correlated with markers of inflammation (i.e., CRP and IL-6).

## DISCUSSION

The novel findings of this study were that *1*) middle-aged/older men with low testosterone have higher circulating concentrations of ET-1 compared with young and age-matched men with normal testosterone; *2*) there was no difference in circulating ET-1 between young and middle-aged/older men with normal testosterone, and *3*) higher circulating ET-1 is associated with reduced endothelial function. However, endothelial cell expression of ET-1 was not different between age or gonadal status groups.

Testosterone concentrations decline gradually with increasing age at a rate of ~10% per decade starting at the age of 30 yr (31); accordingly, data from the Baltimore Longitudinal Study of Aging indicate that 30% of men over 70 yr and 50% of men over 80 yr have low serum testosterone (32). It is important to note that low serum testosterone alone does not meet the definition of “androgen deficiency,” which includes signs and symptoms such as reduced libido, erectile dysfunction, depressed mood, poor concentrations and memory, and/or increased fatigue (33). Regardless, the biological effects of low testosterone, even without signs and/or symptoms of androgen deficiency, manifest in increased cardiovascular disease risk (2-6). For example, we demonstrated lower FMD among middle-aged/older men with low testosterone compared with age-matched men with testosterone levels ≥400 ng/dL (7), suggesting that low testosterone [i.e., testosterone <300 ng/dL as defined by American Urological Association (34)] accelerates age-related declines in vascular endothelial function. However, the mechanisms by which low testosterone contributes to this reduction in vascular function have yet to be fully elucidated.

In our previous study reporting a greater age-associated vascular endothelial dysfunction in men with low testosterone, we demonstrated an improvement in FMD during an acute infusion of ascorbic acid and associations with proinflammatory cytokines, suggesting that increased oxidative stress and inflammation contribute to the exaggerated age-associated reduction in endothelial function observed in men with low testosterone (7). However, the ascorbic acid infusion did not rescue FMD levels to youthful levels and correlations were moderate, explaining ~30% of the variance in FMD, indicating that other mechanisms likely contribute to the differences in FMD. Therefore, we sought to identify other cellular mechanisms that contribute to the accelerated vascular endothelial dysfunction observed in this cohort. In this regard, ET-1 is the most potent endogenous vasoconstrictor known and is primarily produced by the endothelial cells and VSMCs (35, 36). ET-1 produced by endothelial cells acts in a paracrine and autocrine fashion, as endothelial cells secrete ET-1 abluminally toward VSMCs (37). Most ET-1 in the circulation is cleared quickly by ETB receptors in the lungs, kidneys, and liver (38, 39), keeping concentrations low; therefore, higher circulating ET-1 may be reflective of increased local release, as is the case in pathological conditions such as atherosclerosis (40), pulmonary hypertension (41), and heart failure (42). Alternatively, impaired clearance of ET-1 may also contribute to higher circulating ET-1 concentrations, however, we were not able to distinguish between increased production versus reduced clearance in the current study. Regardless, previous studies have demonstrated that older men have greater ET-1 expression in both the circulation and in vascular endothelial cells compared with young men (9). Further, use of the selective ETA receptor inhibitor BQ-123 has revealed a greater vasoconstrictor response to ET-1 in older men (9, 12, 13). Together, these studies have led to the conclusion that elevated ET-1 contributes to age-associated reductions in vascular endothelial function. However, these studies did not consider the gonadal status of participants.

We did not observe group differences in circulating ET-1 between young and middle-aged/older men with normal (i.e., ≥400 ng/dL) testosterone, whereas middle-aged/older men with low testosterone have greater circulating ET-1. Together with the inverse association between ET-1 and FMD (Fig. 2), it appears likely that ET-1 may be a mechanism by which low testosterone accelerates vascular aging in middle-aged/older men. To gain additional insight into the potential confounding effects of age on the association between circulating ET-1 and FMD, we used partial correlation analyses to partial out the effects of age from testosterone. We found that the association between ET-1 and FMD weakened when controlling for age, however controlling for testosterone reduced this association so that it was no longer significant. Collectively, these findings suggest that low testosterone contributes to accelerated vascular aging in middle-aged/older men, at least in part via its effects on the ET-1 system, and highlights the need to evaluate gonadal status in studies that include middle-aged/older men.

We observed no differences in endothelial ET-1 expression in either arterial or venous endothelial cells between age groups or by gonadal status, raising the possibility that higher circulating ET-1 in middle-aged/older men with low testosterone comes from nonendothelial sources. These nonendothelial sources of ET-1 include vascular smooth muscle cells and immune cells such as monocytes, neutrophils, and mast cells (8, 43). Considering that circulating proinflammatory cytokines (IL-6, CRP) are elevated, it is possible that the higher concentrations of ET-1 observed in men with low testosterone arise from immune cells. Consistent with this hypothesis, we observed higher white blood cell counts in middle-aged/older men with low testosterone, however, there were no differences in either monocytes or neutrophils. Similarly, there were no associations between ET-1 concentrations and any immune cells measured in this study. The source of higher circulating levels of ET-1 in middle-aged/older men with low testosterone remains unclear; other immune cells (e.g., mast cells), vascular smooth muscle cells, and endothelial cells remain possibilities. Although we did not observe differences in ET-1 within the endothelial cells, a limitation of endothelial staining for ET-1 concentrations is that ET-1 is rapidly trafficked out of endothelial cells upon synthesis (37). Therefore, it is possible that these intracellular concentrations may not be an accurate representation of endothelial ET-1 synthesis. More research is needed to fully elucidate the causes of greater circulating ET-1 levels in this cohort.

Surprisingly, we observed no associations between ET-1 and insulin concentrations or HOMA-IR. It is well documented in both animal models (44) and humans (45) that insulin-stimulated production and secretion of ET-1 from endothelial cells. Further, this effect is exaggerated with insulin resistance (45, 46). In this study, middle-aged and older men with low testosterone were more insulin resistant (estimated via HOMA-IR) and had ~2× higher insulin concentrations compared with young and age-matched men with normal testosterone. However, insulin concentrations were still relatively low and we excluded participants with diabetes or serum glucose >126 mg/dL, so it is possible that insulin concentrations did not reach a threshold at which they contribute to the production of ET-1 in these participants. Other confounding factors including higher BMI and triglycerides may have also had an impact. There was a significant association between BMI and ET-1 (Fig. 3), as well as an inverse association between BMI and FMD. These associations are consistent with previously reported links between obesity and increased ET-1 (47-49) and may contribute to the increased ET-1 concentrations in middle-aged/older men with low testosterone. Because increased age and low testosterone are generally associated with worse cardiometabolic health and a higher BMI, we used partial correlation analysis to determine whether age and/or testosterone were contributing to the associations between BMI and ET-1. We found that controlling for age weakened this association, BMI and ET-1 remained significantly correlated. In contrast, controlling for testosterone reduced the association between BMI and ET-1 such that they were no longer significantly correlated. Therefore, it appears as though low testosterone may contribute to the effects of higher BMI on increased ET-1 that has been reported previously (49). However, these associations should be interpreted with caution as we are not able to completely isolate the effects of BMI from those of low testosterone in the current cohort; future studies among lean men with low testosterone or models of testosterone suppression (e.g., androgen deprivation therapy for prostate cancer) should further investigate how ET-1 is regulated by testosterone.

These data regarding the effects of gonadal status on circulating ET-1 in older men are similar to previous reports from women and female animals. Estrogen-deficient postmenopausal women have higher circulating ET-1 concentrations compared with premenopausal women (8) and postmenopausal women had decreased plasma ET-1 following 6 mo of estrogen-based hormone therapy (50). In addition, ovariectomized rats (i.e., low estradiol) have higher circulating ET-1, which is attenuated via estradiol add-back (51). Together with the data in the present manuscript, we support previous research asserting a sex-specific role of sex hormones in regulating the ET system (8, 52).

## EXPERIMENTAL CONSIDERATIONS AND LIMITATIONS

We did not measure ETA or ETB receptor density on either endothelial cells or VSMCs. The inclusion of these measures would add insight into whether changes in receptor density and/or sensitivity contribute to the effects of higher ET-1 concentrations on FMD observed in this study. However, at least one previous report indicates that ETB receptor expression was not different between young and older men despite lower testosterone concentrations in older men (53). In contrast, no study that we are aware of has assessed age-related changes in ETA receptor density or sensitivity. Several studies have used ETA receptor-specific inhibition with BQ-123 and determined that older men have greater ETA-mediated vasoconstriction compared with younger men (12, 13). Whether those age-related differences are due to increased ETA receptor density/sensitivity, increased circulating ET-1, or another mechanism is not clear at this time.

Aldosterone and mineralocorticoid receptors play a role in regulating ET-1 expression and sensitivity (54) and there is some evidence that testosterone downregulates aldosterone production (55). Therefore, low testosterone may allow for higher aldosterone production which could be a mechanism for the increased ET-1 that we observed in this study. Unfortunately, we did not measure aldosterone or mineralocorticoid receptor expression in this study and are not able to provide additional insight.

The cross-sectional study design prevents us from drawing definitive conclusions regarding the cause-and-effect nature of higher circulating ET-1 on lower FMD in middle-aged/older men with low testosterone. Finally, only men included in this study were relatively healthy and thus, the findings may not be generalizable to men with preexisting cardiovascular conditions.

## Conclusions

In conclusion, we demonstrate that higher plasma ET-1 in middle-aged/older men with low testosterone contributes to accelerated age-associated reductions in endothelial function. In addition, we did not observe differences in endothelial cell expression of ET-1, suggesting that higher circulating ET-1 in middle-aged/older men with low testosterone may arise from non-endothelial cell sources, such as monocytes, neutrophils, mast cells, and or vascular smooth muscle cells. These data are important in enhancing our understanding of how low testosterone contributes to vascular endothelial dysfunction and increased CVD risk in older men.

## Figures and Tables

**Table 1.** Participant characteristics

Variable	Young Men	MA/O Men with Normal T	MA/O Men with Low T	*P* Value
N	20	20	20	
Race	16 C, 3 A, 1 M	19 C, 1 AA	19 C, 1 AA	
Age, yr	30±4	59 ± 6*	60 ± 8*	<0.001
Mass, kg	78.0 ± 10	87.4 ± 13.7*	96.9 ± 16*	<0.001
Height, cm	181.1 ± 6.5	178.0 ± 7.1	177.2 ± 7.6	0.182
BMI, kg/m^2^	23.8 ± 2.8	27.5 ± 3.7*†	30.8 ± 4.5*	<0.001
Body fat, %	22.9 ± 7.4	29.4 ± 4.9*	31.7 ± 4.9*	<0.001
Systolic BP, mmHg	117.8 ± 9.3	126.6 ± 7.9*	129.1 ± 8.3*	<0.001
Diastolic BP, mmHg	73.3 ± 5.2	78.9 ± 8.0*	81.1 ± 7.1*	0.002
$\dot{V}o_{2\max}$, mL/kg/min	44.9 ± 9.3	30.7 ± 5.5*	27.2 ± 4.9*	<0.001
Total cholesterol, mg/dL	152.8 ± 28.0	181.8 ± 50.2*	187.3 ± 27.7*	0.009
LDL cholesterol, mg/dL	107.8 ± 30.2	134.7 ± 46.3	143.4 ± 29.0*	0.009
Triglycerides, mg/dL	74.4 ± 30.2	84.8 ± 36.2†	133.8 ± 63.4*	<0.001
Glucose, mg/dL	87.0 ± 8.9	89.0 ± 6.6†	95 ± 6.5*	0.003
Insulin, uIU/mL^a^	3.0 (2.0–4.0)	3.0 (2.0–4.0)†	6.0 (2.5–8.0)*	0.002
HOMA-IR^a^	0.63 (0.39–0.95)	0.67 (0.47–0.92)†	1.47 (0.59–1.96)*	0.001
Testosterone, ng/dL	510 ± 63	512 ± 115†	265 ± 47*	<0.001
Free testosterone, ng/dL	11.5 ± 3.5	8.8 ± 1.5*†	6.7 ± 1.9*	<0.001
Estradiol, pg/mL	33.0 ± 11.8	46.7 ± 15.2*	36.1 ± 16.6	0.012
Follicle stimulating hormone, mIU/mL^a^	3.10 (1.98–4.30)	5.35 (3.23–6.43)	5.40 (3.25–7.43)*	0.04
Luteinizing hormone, mIU/mL	3.35 ± 1.25	3.72 ± 1.00	3.54 ± 3.03	0.840
Sex hormone binding globulin, nmol/L	26.5 ± 9.9	48.6 ± 14.0*†	26 ± 9.9	<0.001

Data were examined using one-way ANOVAs and are displayed as means ± SD. Non-normally distributed data (^a^) were natural log-transformed before statistical examination and are displayed as median (interquartile range) in their original units. A, Asian; AA, African American; BMI, body mass index; BP, blood pressure; C, Caucasian; HOMA-IR, homeostatic model analysis of insulin resistance; LDL, low-density lipoprotein; M, more than one race; MA/O, middle-aged/older; T, testosterone. *P < 0.05 compared with younger men, †*P* < 0.05 compared with MA/O men with low T.

**Table 2.** Endothelial cell ET-1 expression and circulating factors

Variable	Young Men	MA/O Men with Normal T	MA/O Men with Low T	*P* Value
*Venous Endothelial Cells*				
*n*	15	17	17	
Venous ET-1/HUVEC intensity	0.990 ± 0.018	0.986 ± 0.015	0.984 ± 0.017	0.616
Venous vWF/HUVEC intensity	0.995 ± 0.017	0.993 ± 0.013	0.991 ± 0.015	0.744
*Arterial Endothelial Cells*				
*n*	12	14	12	
Arterial ET-1/HUVEC intensity	0.996 ± 0.016	0.993 ± 0.019	0.984 ± 0.02	0.222
Arterial vWF/HUVEC intensity	1.000 ± 0.013	0.996 ± 0.019	0.994 ± 0.017	0.677
*Circulating Factors*				
Oxidized LDL, U/L	67.50 ± 29.8	73.3 ±23.0	72.4± 14.2	0.710
Total antioxidant status, mmol/L^a^	1.74 (1.70–1.85)	1.70 (1.62–1.76)	1.73 (1.65–1.83)	0.496
Interleukin-6, pg/mL^a^	0.74 (0.59–1.16)	1.40 (1.06–1.77)*†	2.05 (1.37–2.97)*	<0.001
C-reactive protein, mg/L^a^	0.47 (0.30–0.61)	0.84 (0.53–1.77)*†	1.61 (1.00–4.30)*	<0.001
White blood cell count, 10 × 9/L^a^	5.05 (4.33–5.60)	4.60 (3.93–5.68)†	6.10 (5.18–6.98)	0.045
Absolute neutrophils, 10 × 9/L	2.81 ± 1.40	2.76 ± 1.08	3.33 ± 1.39	0.323
Neutrophils, %^a^	8.15 (6.60–10.50)	9.20 (8.20–11.00)	9.05 (7.23–11.98)	0.737
Absolute monocytes, 10 × 9/L^a^	0.40 (0.40–0.50)	0.40 (0.30–0.60)	0.50 (0.40–0.80)	0.469
Monocytes (%)^a^	53.55 (45.55–59.78	57.50 (51.10–61.70)	55.15 (43.80–60.60)	0.369

Data were examined using one-way ANOVAs and are displayed as means ± SD. ET-1, endothelin-1; HUVEC, human umbilical vein endothelial cell; LDL, low-density lipoprotein; MA/O, middle-aged/older; T, testosterone; vWF, von Willebrand Factor. *P < 0.05 compared with younger men, †*P* < 0.05 compared with MA/O men with low T, ^a^non-normally distributed data that were natural log transformed before analysis.

**Table 3.** Flow-mediated dilation

Variable	Young Men	MA/O Men with Normal T	MA/O Men with Low T	*P* Value
Baseline brachial artery diameter, mm	4.10 ± 0.51	4.65 ± 0.63*†	5.02 ± 0.52*	<0.001
Brachial artery diameter change, mm	0.31 ± 0.07	0.22 ± 0.10*	0.19 ± 0.09*	<0.001
FMD, %	7.3 ± 1.3	5.7 ± 2.2*†	4.0 ± 1.8*	<0.001

Data were examined using one-way ANOVAs and are displayed as means ± SD. FMD, Flow-mediated dilation; MA/O, middle-aged/older; T, testosterone. *P < 0.05 compared with younger men, †*P* < 0.05 compared with MA/O men with low T.

**Table 4.** Correlation matrix

Variable	Total Testosterone, ng/dL		ET-1, pg/mL^a^		FMD, %	
	*r*	*P*	*r*	*P*	*r*	*P*
Age, yr	**−0.409**	**0.001**	**0.359**	**0.005**	**−0.461**	**<0.001**
Systolic BP, mmHg	**−0.306**	**0.017**	**0.489**	**<0.001**	**−0.373**	**0.004**
Diastolic BP, mmHg	**−0.339**	**0.008**	**0.355**	**0.006**	**−0.294**	**0.024**
BMI, kg/m^2^	**−0.544**	**<0.001**	**0.396**	**0.002**	**−0.395**	**0.002**
Total testosterone, ng/dL			**−0.483**	**<0.001**	**0.486**	**<0.001**
Free testosterone, ng/dL	**0.467**	**<0.001**	**−0.353**	**0.006**	**0.421**	**<0.001**
Estradiol, pg/mL	**0.376**	**0.003**	−0.047	0.723	0.006	0.964
Follicle-stimulating hormone, mIU/mL^a^	−0.201	0.124	0.134	0.310	−0.090	0.499
Luteinizing hormone, mIU/mL^a^	0.201	0.123	0.145	0.273	0.219	0.096
Sex hormone binding globulin, nmol/L^a^	**0.521**	**<0.001**	−0.107	0.421	0.192	0.144
Insulin, uIU/mL^a^	**−0.372**	**0.004**	−0.003	0.984	−0.203	0.124
HOMA-IR^a^	**−0.350**	**0.006**	0.019	0.884	−0.188	0.154
ET-1, pg/mL^a^	**−0.448**	**<0.001**			−**0.371**	**0.004**
IL-6, pg/mL^a^	**−0.462**	**<0.001**	**0.387**	**0.003**	**−0.461**	**<0.001**
CRP, mg/L^a^	**−0.550**	**<0.001**	**0.505**	**<0.001**	**−0.396**	**0.002**
TAS, mmol/L^a^	−0.068	0.607	−0.092	0.490	0.052	0.699
Oxidized LDL, U/L^a^	0.070	0.605	0.097	0.477	0.005	0.969
WBC^a^	**−0.347**	**0.007**	0.038	0.773	−0.084	0.526
Neutrophils, ×109/L^a^	**−0.271**	**0.038**	0.078	0.560	−0.101	0.452
Monocytes, ×109/L^a^	−0.160	0.225	−0.004	0.978	−0.045	0.736
FMD, %	**0.486**	**<0.001**	**−0.414**	**0.001**	–	–

Data were examined using Pearson correlation analyses. Non-normally distributed data (^a^) were natural log transformed before statistical examination. Bold text indicates significant correlations. BMI, body mass index; BP, blood pressure; HOMA-IR, homeostatic model assessment for insulin resistance; LDL, low-density lipoprotein; T, testosterone; WBC, white blood count.

## Data Availability

Data will be made available upon reasonable request.
